# Gepta-EX: a multi-channel germanium detector for X-ray absorption fine structure

**DOI:** 10.1107/S1600577526004261

**Published:** 2026-05-21

**Authors:** Abdul K. Rumaiz, Francesca Capocasa, Anthony J. Kuczewski, Giovanni Pinaroli, Thomas Krings, Bruce Ravel, D. Peter Siddons

**Affiliations:** a NSLS II Brookhaven National Laboratory Upton NY11973 USA; b Instrumentation Department Brookhaven National Laboratory Upton NY11973 USA; c Forschungszentrum GmbH, 52425Jülich, Germany; d National Institute of Standards and Technology Gaithersburg MD20899 USA; Paul Scherrer Institut, Switzerland

**Keywords:** EXAFS, germanium, detector, X-ray absorption, spectroscopy

## Abstract

Fluorescence-mode X-ray absorption spectroscopy (XAS) at high photon energies requires detectors with high stopping power and excellent energy resolution to measure weak element-specific signals. Traditional silicon-based detectors suffer from poor efficiency above ∼20 keV, while most high-*Z* materials such as cadmium telluride, cadmium zinc telluride, and gallium arsenide have focused predominantly on imaging applications with limited spectroscopic resolution. In contrast, germanium combines excellent stopping power with superior intrinsic energy resolution due to its low Fano factor and favorable charge transport properties, making it ideal for high-resolution spectroscopy in the 15 keV to 100 keV range. To address these requirements, we report on the development, fabrication, and performance evaluation of Gepta-EX, a compact multi-channel high-purity germanium (HPGe) detector system for fluorescence-mode XAS. The Gepta-EX system features a monolithic seven-channel HPGe pixel array integrated with low-noise CUBE charge-sensitive preamplifiers, housed within a thermally isolated compact cryostat operating near 90 K. The detector achieves energy resolutions of 218 eV at 5.9 keV (from a ^55^Fe source) and 373 eV at 59.5 keV (from a ^241^Am source), with uniform performance across all channels. By avoiding the escape peak interference commonly associated with silicon detectors and providing stable, high-resolution performance, Gepta-EX represents a powerful tool for high-energy X-ray spectroscopy.

## Abstract 2

Gepta-EX is a compact seven-channel high-purity germanium detector designed to deliver high-resolution fluorescence X-ray absorption fine-structure measurements at photon energies where silicon detectors become effectively transparent. It provides excellent energy resolution across a broad energy range, avoids silicon escape-peak artifacts, and offers uniform multi-channel performance, enabling reliable high-energy X-ray absorption studies of advanced materials.

## 1. Introduction

X-ray absorption fine structure (XAFS) spectroscopy is a cornerstone analytical technique at synchrotron light sources, widely employed for investigating the local atomic and electronic structure of matter across a broad range of materials (Newville, 2014 ▸). By scanning the incident X-ray energy across an absorption edge and monitoring the attenuation of the direct beam or the emission secondary photons, XAFS provides element-specific insight into oxidation states, bond distances, and coordination environments. In the standard ‘transmission mode’, XAFS measurements are conducted using ionization chambers to record the incident and transmitted beam intensities through a sample. While effective for concentrated and homogeneously thick samples, this mode becomes impractical for dilute systems, thin films, or heterogeneous materials where the signal-to-noise ratio is inherently low. In such cases, XAFS is performed in fluorescence mode, where the X-ray fluorescence emitted by the sample is measured. This often uses energy-dispersive detectors capable of isolating the element-specific emission signal from background and elastic scattering. Silicon drift detectors (SDDs) have been the workhorse detectors for fluorescence-mode XAFS due to their high energy resolution, compact design, and fast readout. However, their detection efficiency deteriorates sharply above ∼15 keV, limiting their effectiveness for studies involving high-*Z* elements or high-energy *K*-edges (*e.g.* 4*d* and 5*d* transition metals, lanthanides, and actinides). In addition, silicon-based detectors are susceptible to escape peaks that may interfere with the detection of fluorescence lines near Si *K*α (∼1.74 keV), further complicating spectral interpretation.

High-purity germanium (HPGe) detectors present an attractive alternative, offering both high energy resolution and significantly improved quantum efficiency at high photon energies. Germanium’s higher atomic number increases its X-ray stopping power, making it particularly well suited for high-energy XAFS measurements. Moreover, the Ge escape peak (*K*α 9.8 keV) lies far from the absorption edge, avoiding interference. These features make germanium an ideal detector material for next-generation XAFS beamlines operating in the 15 keV to 100 keV range. While significant effort has focused on the development of SDD arrays, comparatively little attention has been given to advancing HPGe detectors for synchrotron applications. HPGe detectors offer superior energy resolution and higher detection efficiency for high-energy photons relative to SDDs, making them particularly well suited for demanding spectroscopic measurements. Commercial HPGe detectors are widely available and provide excellent energy resolution for high-energy X-rays. However, standard systems are typically designed for low to moderate count-rate applications and often lack the readout speed, array scalability, and real-time photon-by-photon processing capabilities required for modern synchrotron experiments. As a result, while they demonstrate the fundamental advantages of HPGe sensors, their use in high-flux, multi-element beamline setups is limited without significant customization. Recent efforts, such as those under the LEAPS-INNOV initiative (Manzanillas *et al.*, 2023 ▸; Goyal *et al.*, 2025 ▸; Goyal *et al.*, 2026 ▸), have begun to address these limitations by developing next-generation HPGe detectors optimized for synchrotron applications, combining fast readout electronics, advanced digital pulse processing, and arrayed sensor geometries. These advances point toward a new class of HPGe systems capable of operating at high flux with high energy resolution, bridging the gap between commercial detectors and state-of-the-art synchrotron requirements.

In this work, we present the development and performance evaluation of Gepta-EX, a seven-channel custom HPGe pixel detector designed for fluorescence-mode XAFS applications. The detector features a segmented germanium sensor coupled with a low-noise commercial charge-sensitive preamplifier (Bombelli *et al.*, 2011 ▸)^1^ for each channel, enabling energy-dispersive readout with excellent resolution and low electronic noise. The system is cryogenically cooled using a compact commercial cryostat that incorporates an integrated cooling unit, significantly reducing the overall footprint and eliminating the need for external cryogen handling. This germanium-based detection platform is particularly well suited for studying the *K*-edges of 4*d* transition metals (*e.g.* Zr, Mo, Rh, Ag) and *L*-edges of 5*d* elements (*e.g.* W, Pt), where traditional silicon-based detectors fall short. By enabling high-quality XAFS measurements at higher energies, this detector opens new possibilities for research in catalysis, photovoltaics, electrochemical energy storage, nuclear waste management, high-performance coatings, and emerging quantum materials.

## 2. Sensor fabrication

The Brookhaven National Laboratory (BNL) group has developed and fabricated a range of germanium strip and pixel detectors systems that are currently deployed at high-energy powder diffraction beamlines (Rumaiz *et al.*, 2014 ▸; Rumaiz *et al.*, 2018 ▸). These detectors use germanium sensors fabricated using a technique pioneered by D. Protic and T. Krings (Krings *et al.*, 2015 ▸), in which pixel isolation is achieved through precision etching of trenches into the detector wafer. This approach enables precise control over segmentation and isolation, making it well suited for high-resolution X-ray detection at high photon energies. These detectors have proven effective in synchrotron applications that require high stopping power and excellent energy resolution, particularly in high-energy diffraction experiments.

**Figure 1:**
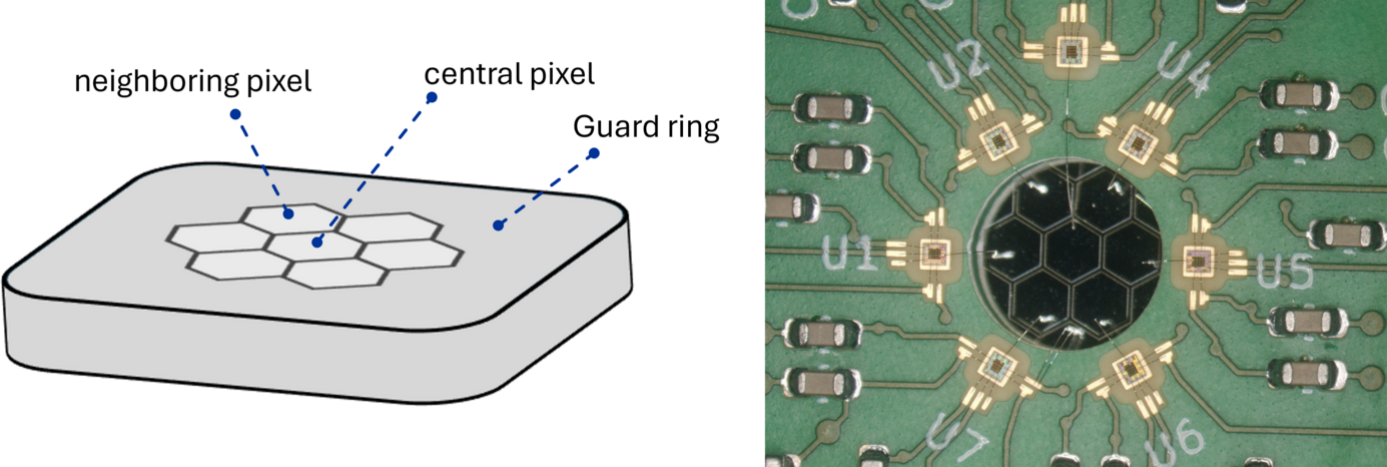
Schematic cross section of the Gepta-EX sensor (left) and zoomed-in photograph of the sensor mounted on the PCB (right).

**Figure 2:**
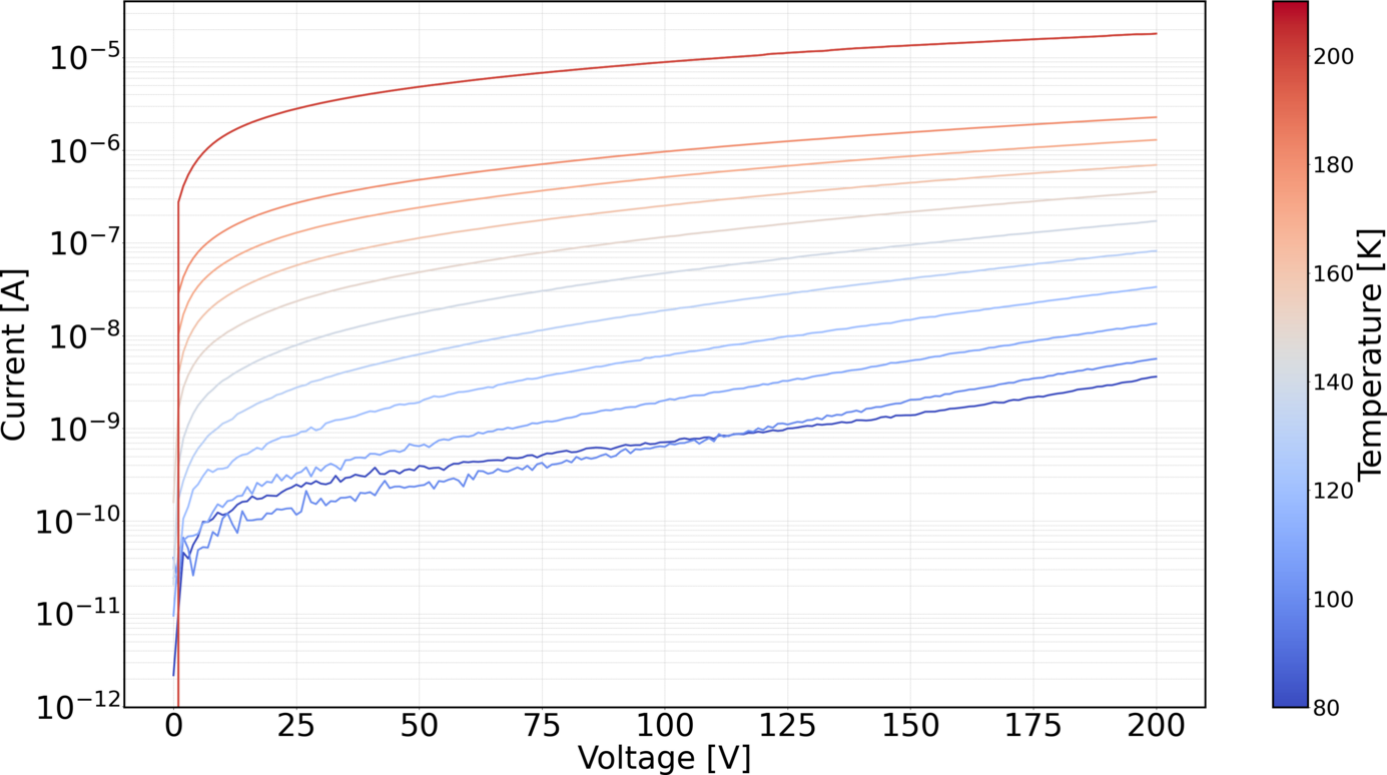
*I*–*V* characteristics of the Gepta-EX sensor at different temperatures. During the measurement, the voltage was swept at the rear n^+^ contact of the sensor while all pixels and the guard ring were grounded. The plot shows the sensor leakage current measured at the back-side contact.

The Gepta-EX sensor was fabricated using an n-type HPGe wafer from Umicore, Belgium. The impurity concentration was lower than 10^10^ cm^−3^. The total area of the sensor is about 12 mm × 12 mm with a thickness of about 3 mm. In this fabrication process, the front side of the Ge wafer is implanted with boron (B) ions to produce a thin p-type layer, which serves as a rectifying junction, while the n^+^ back contact is formed using a thermally evaporated amorphous germanium (a-Ge) layer that acts as an ohmic contact. A thin aluminium film is then deposited on both sides of the wafer. The front side is photolithographically patterned to define the individual pixels. The aluminium pattern also serves as a mask during plasma etching, allowing trenches to be etched between pixels to ensure electrical isolation. The sensor array consists of a monolithic seven-hexagonal-pixel configuration with 1.7 mm pixels. A photograph of the sensor is shown in Fig. 1 ▸. All seven channels share the same bias voltage from the back side. The device was diced and mounted on a cryogenic probe station for electrical characterization. Fig. 2 ▸ shows the leakage current measured at the sensor’s back-side contact as a function of temperature.

## 3. Detector system

**Figure 3:**
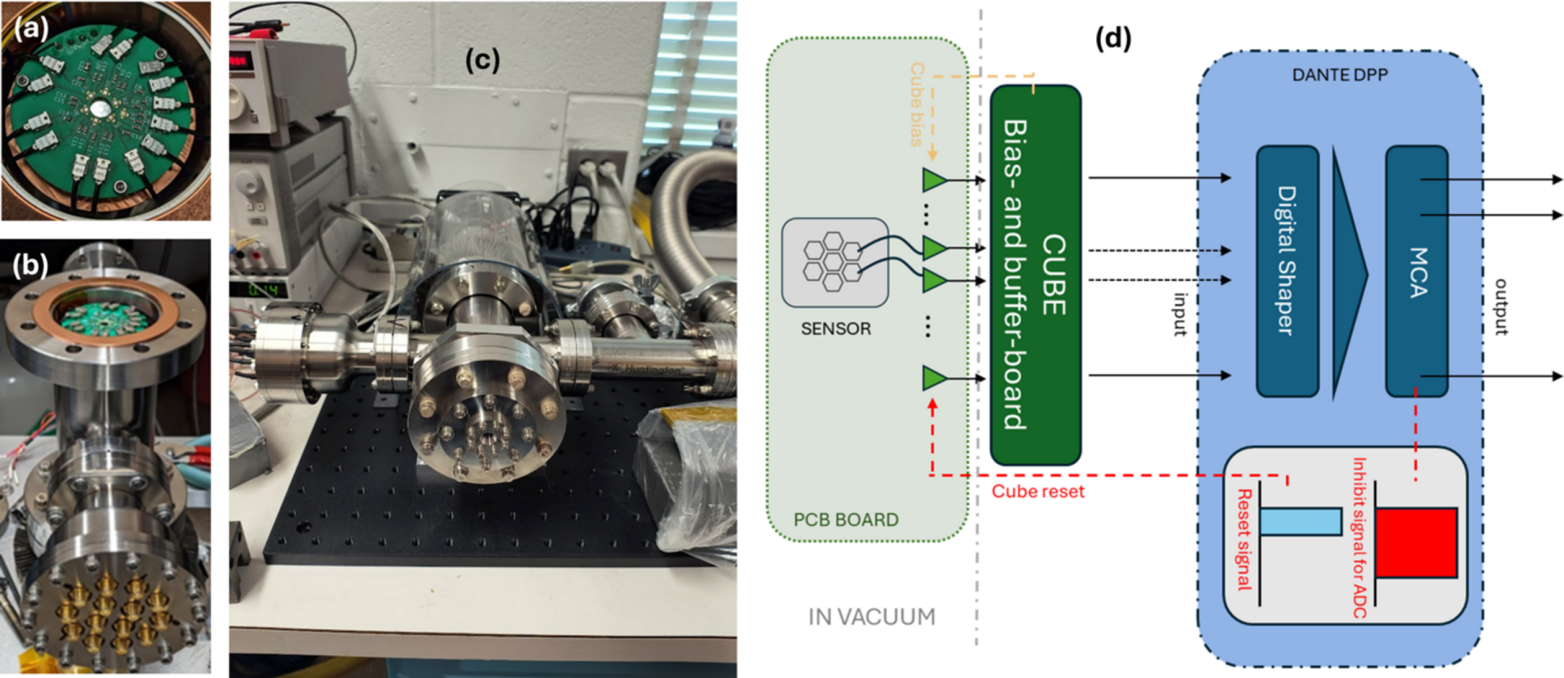
(*a*) Sensor wire bonded to the CUBE. (*b*) Detector with custom vacuum feed-through. (*c*) Full detector vacuum housing. (*d*) Block diagram of the detector system.

Fig. 3 ▸ shows a block diagram and photographs of the seven-channel germanium detector array wired to a custom printed circuit board (PCB) equipped with CUBE charge-sensitive preamplifiers. The PCB is mounted on the cryostat assembly via a thermally isolated support post, minimizing conductive heat transfer to the cold stage. The germanium sensor itself is mounted on a copper cold plate, which is thermally anchored to a Stirling cycle cryocooler (SunpowerInc, 2025 ▸). This compact closed-cycle cooling system eliminates the need for liquid cryogens and provides stable cryogenic temperatures near 100 K. The cryocooler is equipped with an active vibration damper on the cold head to suppress mechanical noise that could otherwise degrade energy resolution during operation.

The bias voltage (+HV) is applied to the unstructured amorphous germanium back contact, while the guard ring surrounding the segmented detector area is held at 2 V potential. The detectors are operated close to liquid nitrogen temperature, ensuring low leakage currents and stable performance. The PCB hosts seven low-noise CUBE preamplifiers (model PRE 039), each connected to a detector segment via wire bonding. These preamplifiers are optimized for pulsed-reset operation with an internal feedback capacitance of 50 fF and designed for detector input capacitances in the range of 3 pF to 10 pF (Bombelli *et al.*, 2012 ▸) closely matching the simulated capacitance of our detector geometry. During operation, the PCB stabilizes at approximately −30°C. Signal and reset lines for preamplifiers are routed through a set of eight small format RF vacuum feed-throughs; power supplies, heater and temperature readout lines are routed to a nine-pin vacuum feed-through. The preamplifier output signals are processed by the DANTE digital pulse processor, which applies trapezoidal pulse shaping for optimal energy resolution and noise performance. This configuration enables high-resolution, low-noise operation suitable for high-energy X-ray spectroscopy applications while maintaining a compact and thermally efficient detector assembly.

## 4. Spectroscopy results

**Figure 4:**
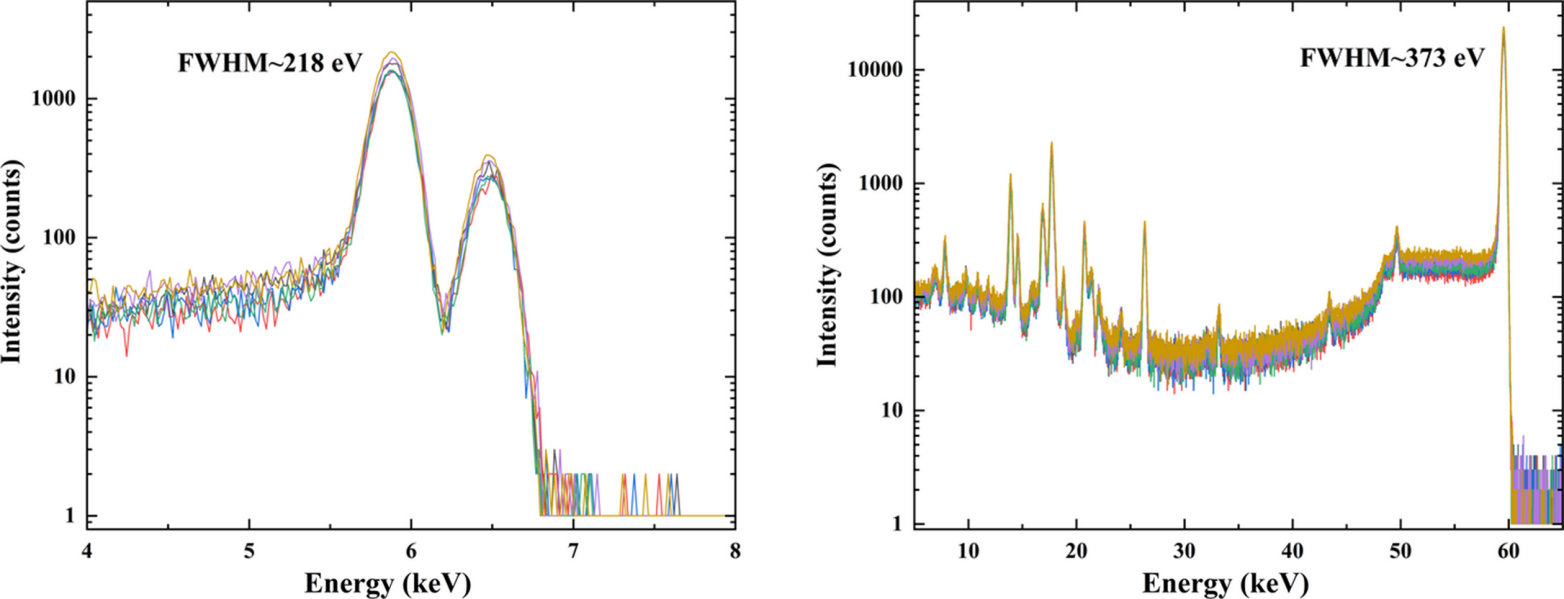
^55^Fe (left) and ^241^Am (right) response for all channels. Measured FWHM are 218 eV and 373 eV for ^55^Fe and ^241^Am, respectively.

The detector was characterized using sealed radioactive sources, with measurements performed without a collimator on the sensor. Fig. 4 ▸ presents the response from six channels when exposed to ^55^Fe and ^241^Am sources. One channel was disconnected due to persistent noise issues. For ^55^Fe, a full width at half-maximum (FWHM) energy resolution of 218 ± 0.9 eV was measured at 5.89 keV. During this measurement, the sensor was maintained at 90 K, and the shaping (peaking) time was set to 480 ns. For the ^241^Am, the measured energy resolution was 373 ± 0.7 eV FWHM at 59.5 keV. The response across all six active channels was uniform in both peak position and resolution, demonstrating consistent performance and minimal channel-to-channel variation, which highlights the robustness of the sensor and readout electronics.

It is well established that the energy resolution of a detector is strongly influenced by the peaking (shaping) time of the signal processing electronics which determines the equivalent input charge noise. Equation (1) models the equivalent noise input charge (ENC) as a function of the main noise contributions in a detector system (Rivetti, 2015 ▸),
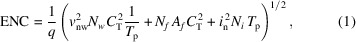
where *C*_T_ is the total capacitance at the input of the front-end electronics, 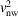 is the power spectral density (PSD) of the series white noise, 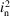 is the PSD of the parallel white noise, *A*_*f*_ is the coefficient associated to the series 1/*f* noise, *T*_p_ is the shaping filter peaking time and *N*_*w*_, *N*_*f*_, *N*_*i*_ are the noise indexes of the shaping filter (De Geronimo & Li, 2011 ▸).

At shorter peaking times, series noise, which is primarily associated with the detector capacitance and the front-end electronics, tends to dominate, resulting in higher ENC and broader spectral peaks. As the peaking time increases, series noise is effectively suppressed, leading to improved charge integration and better energy resolution. However, this improvement comes with trade-offs: at longer peaking times, parallel noise, mainly driven by detector leakage current, becomes more prominent. This can limit further gains in resolution or even degrade performance if the leakage is significant. Additionally, a larger peaking time also affects the maximum event rate which can be processed by the detector.

**Figure 5:**
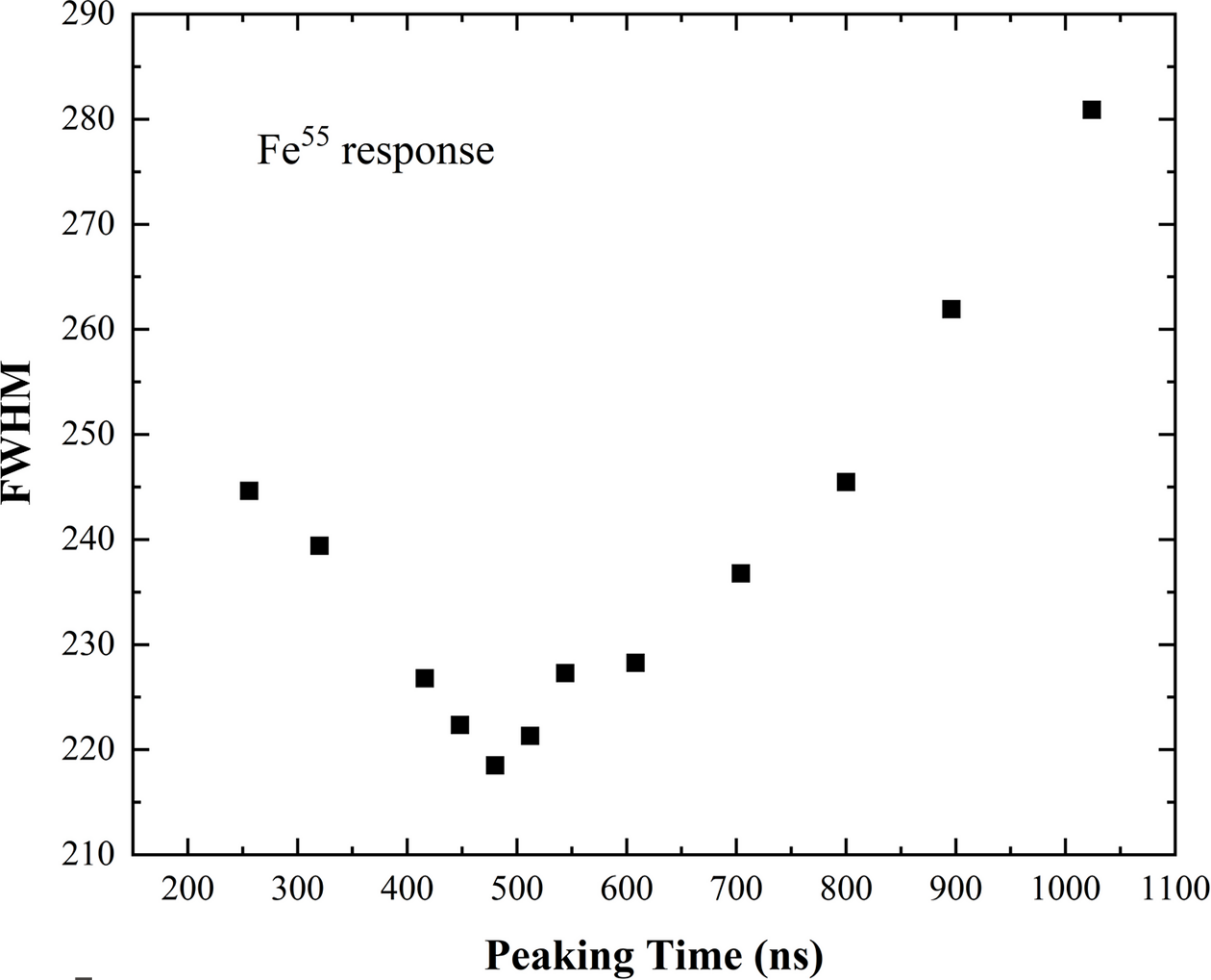
FWHM for the 5.9 keV line from ^55^Fe measured at different peaking time.

Fig. 5 ▸ presents the measured FWHM for the 5.9 keV line from ^55^Fe for a representative channel, as a function of peaking time. The curve shows a characteristic minimum (about 480 ns), where the competing noise contributions (series and parallel) are optimally balanced. This behavior is consistent with theoretical expectations for semiconductor detectors, where the total noise is a combination of series, parallel, and low-frequency (flicker) noise components. While this relatively short optimum peaking time suggests the presence of higher leakage current which contributes to an increased parallel noise, it also indicates that the detector is capable of handling higher photon flux without significant pulse pile-up. With the optimized peaking time of 480 ns the detector system is expected to process up to ∼750k photons s^−1^. Although the spectra presented in Fig. 4 ▸ were acquired at low count rates to characterize intrinsic performance, the peaking-time study demonstrates that the detector can operate at significantly higher throughput with controlled and predictable resolution degradation. The results highlight the importance of carefully tuning the peaking time to match the detector’s intrinsic noise characteristics and operating conditions for optimal spectroscopic performance.

**Figure 6:**
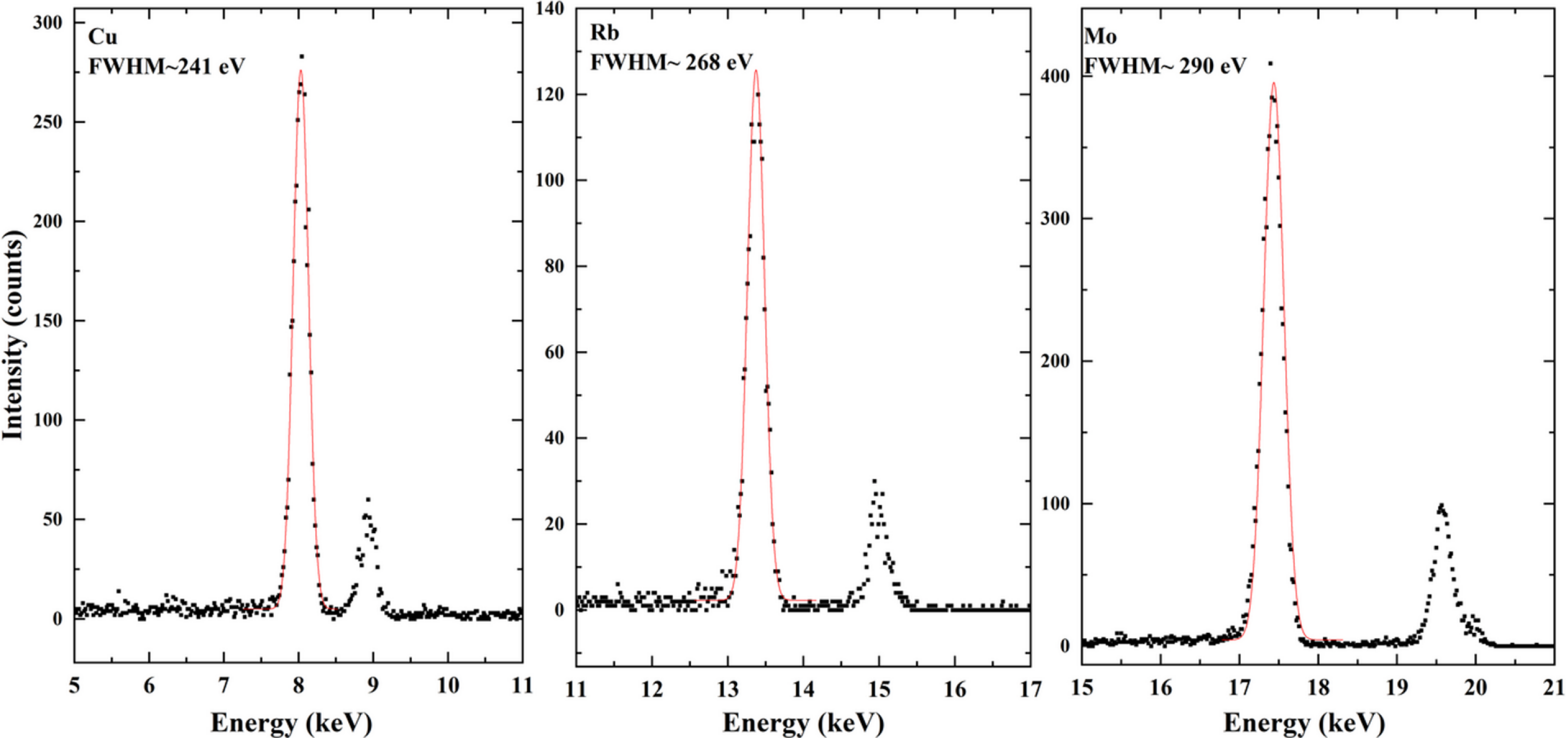
*K*α emission of copper (Cu), rubidium (Rb), and molybdenum (Mo). Measured FWHM are 241 eV, 268 eV, and 290 eV for Cu, Rb, and Mo, respectively.

Fig. 6 ▸ shows *K*α emission of copper (Cu), rubidium (Rb), and molybdenum (Mo) obtained by irradiating metal foils with a ^241^Am source. The measured FWHM are 241 ± 0.9, 268 ± 1, and 290 ± 0.9 for Cu, Rb, and Mo, respectively, indicating good energy resolution and excellent performance across this energy range.

**Figure 7:**
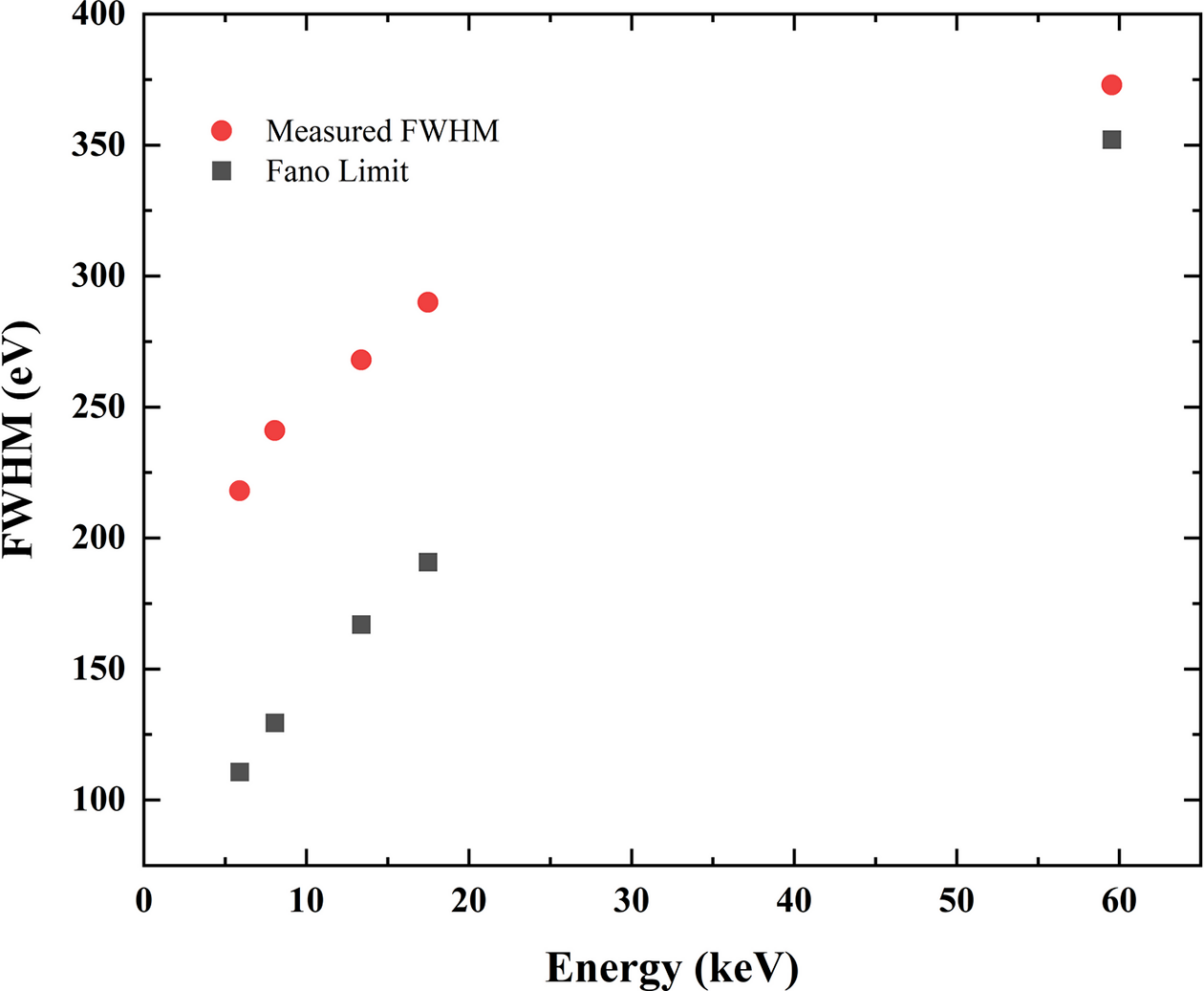
Measured energy resolution compared with the Fano-limited resolution for germanium. Deviations at low energies are due to electronic noise; convergence at higher energies indicates near-intrinsic detector performance.

Fig. 7 ▸ presents a comparison between the measured energy resolution at various photon energies and the theoretical Fano-limited resolution for germanium. The Fano limit represents the fundamental statistical lower bound on energy resolution, determined by the inherent fluctuations in charge carrier generation (Fano, 1947 ▸). As expected, the measured resolutions exceed the Fano limit across the energy range due to additional contributions from system-level noise sources including electronic noise from the readout circuit, charge trapping within the detector bulk, and spatial variations in charge collection efficiency (Bertuccio & Mele, 2023 ▸). In this system, a charge-sensitive preamplifier with an intrinsic noise contribution of approximately 20 electrons was used, contributing to the overall resolution. Despite these practical limitations, the overall trend of the measured data closely follows the shape of the Fano-limited curve, suggesting that the detector operates near its theoretical limit, particularly at higher photon energies. At lower energies, deviations are more pronounced, which is consistent with the increasing relative influence of system noise in this regime. At higher energies, the resolution improves and approaches the Fano curve, indicating that the dominant broadening mechanism becomes statistical in nature, governed by the number of electron–hole pairs generated per incident photon.

## 5. X-ray absorption results

The Gepta-EX detector was tested at the NIST Beamline for Materials Measurements (BMM), beamline 6-BM at the National Synchrotron Light Source II (NSLS-II), Brookhaven National Laboratory, USA. BMM uses a three-pole wiggler source delivering light to a collimating mirror with an Rh-on-Pt coating (Marcus *et al.*, 2004 ▸) upstream of a Si(111) double-crystal monochromator. A toroidal mirror with the same Rh-on-Pt coating focuses the beam to a small spot of about 300 µm FWHM with long tails rejected with slits. XAFS data were collected on a platinum–zirconium alloy codeposited on a silica wafer. The wafer was mounted upright and at a 45° angle relative to the incident beam. As the Gepta-EX detector system was not fully integrated with the beamline data acquisition software, measurements were performed by moving the monochromator stepwise through the energy range of the XAFS spectrum while recording XRF spectra from the Gepta-EX detector at each energy point. Signals from the Zr *K*α emission line were extracted from this sequence of XRF spectra to make an XAFS spectrum. For comparison, data were collected on the same spot on the wafer using a commercial SDD.

**Figure 8:**
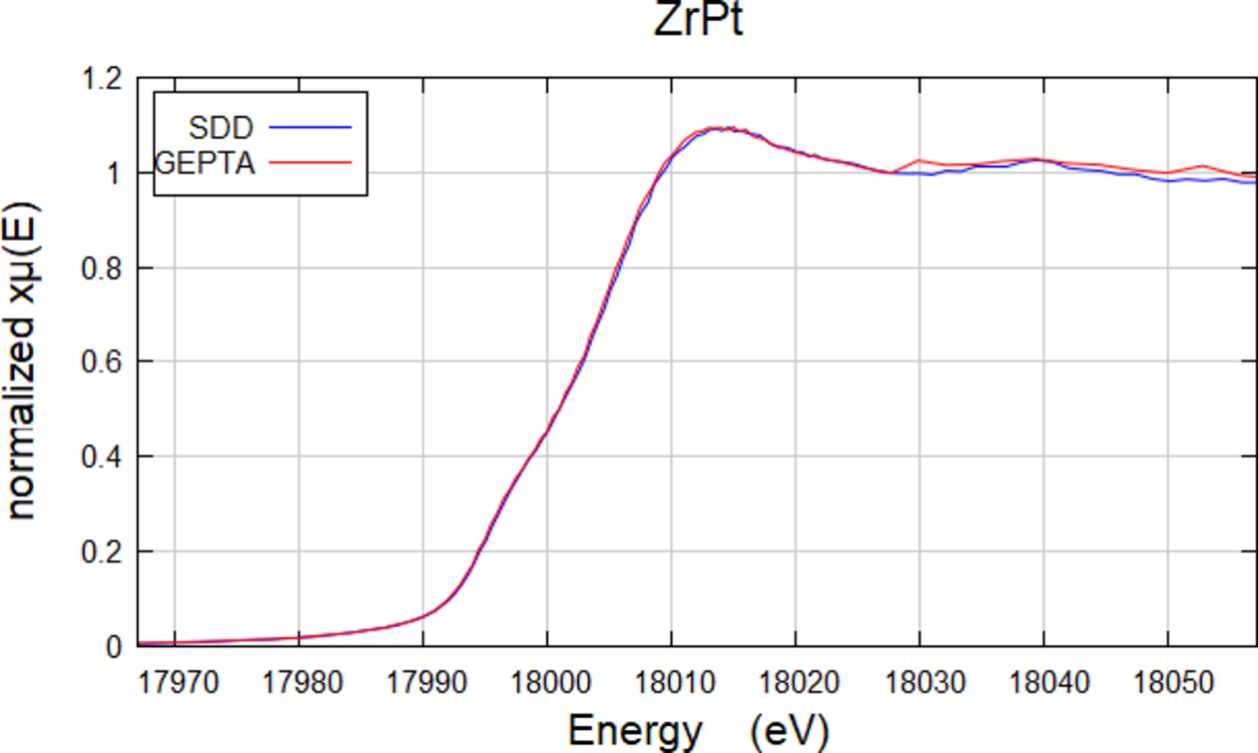
Zr *K*-edge XAFS measured by Gepta-EX and SDD.

Fig. 8 ▸ shows the Zr edge XAFS of the ZrPt sample measured by both detectors. The near-edge features are in  close agreement, demonstrating consistent performance between the Gepta-EX and the SDD. However, some differences were observed in the extended absorption region. These discrepancies indicate systematic uncertainty due to the measurements of the incident beam intensity (*I*_0_) and the Gepta-EX spectra being made during different time windows as a result of the lack of direct integration with the beamline controls. Proper integration of the Gepta-EX is expected to improve the accuracy and consistency of XAFS measurements.

## 6. Conclusions

We have developed, fabricated, and characterized Gepta-EX, a compact multi-channel high-purity germanium detector optimized for fluorescence-mode X-ray absorption spectroscopy at high photon energies. This detector addresses the critical need for high-resolution spectroscopic systems capable of operating effectively beyond the energy range where conventional silicon detectors become transparent and ineffective. Furthermore, the use of germanium eliminates the problem of interference of the escape peak in absorption spectra commonly encountered in silicon-based detectors. The current system is equipped with low-noise CUBE charge-sensitive preamplifiers and integrated into a compact cryostat design, enabling stable cryogenic operation and achieving excellent energy resolution measured as low as 218 eV at 5.9 keV and 373 eV at 59.5 keV under low flux conditions. Upcoming iterations will incorporate a charge-sensitive amplifier featuring significantly lower intrinsic noise. This is expected to further reduce the equivalent noise charge, thereby enhancing the detector’s energy resolution, particularly at lower photon energies where electronic noise currently dominates.

## Data Availability

Data supporting the research findings are not publicly accessible but can be obtained by contacting the authors directly.

## Footnotes

1 Mention of specific commercial products or instruments in this publication is solely for the purpose of providing a clear description of experimental or test procedures. This mention does not constitute an endorsement or recommendation of those specific products by NIST and NSLSII. It also does not imply that the mentioned products are the best available for the intended purpose.
